# Non-invasive measurements of blood glucose levels by time-gating mid-infrared optoacoustic signals

**DOI:** 10.1038/s42255-024-01016-9

**Published:** 2024-03-27

**Authors:** Nasire Uluç, Sarah Glasl, Francesca Gasparin, Tao Yuan, Hailong He, Dominik Jüstel, Miguel A. Pleitez, Vasilis Ntziachristos

**Affiliations:** 1 Institute of Biological and Medical Imaging, Helmholtz Zentrum München, Neuherberg, Germany; 2 Chair of Biological Imaging at the Central Institute for Translational Cancer Research (TranslaTUM), School of Medicine and Health, Technical University of Munich, Munich, Germany; 3 Institute of Computational Biology, Helmholtz Zentrum München, Neuherberg, Germany; 4 DZHK (German Centre for Cardiovascular Research), partner site Munich Heart Alliance, Munich, Germany

**Keywords:** Optical spectroscopy, Sensors and probes, Metabolism

## Abstract

Non-invasive glucose monitoring (NIGM) represents an attractive alternative to finger pricking for blood glucose assessment and management of diabetes. Nevertheless, current NIGM techniques do not measure glucose concentrations in blood but rely on indirect bulk measurement of glucose in interstitial fluid, where glucose is diluted and glucose dynamics are different from those in the blood, which impairs NIGM accuracy. Here we introduce a new biosensor, termed depth-gated mid-infrared optoacoustic sensor (DIROS), which allows, for the first time, non-invasive glucose detection in blood-rich volumes in the skin. DIROS minimizes interference caused by the stratum corneum and other superficial skin layers by time-gating mid-infrared optoacoustic signals to enable depth-selective localization of glucose readings in skin. In measurements on the ears of (female) mice, DIROS displays improved accuracy over bulk-tissue glucose measurements. Our work demonstrates how signal localization can improve NIGM accuracy and positions DIROS as a holistic approach, with high translational potential, that addresses a key limitation of current NIGM methods.

## Abstract (web summary)

A new methodology uses intravital time-gated mid-infrared optoacoustic signals for accurate non-invasive measurements of glucose concentrations in blood-rich volumes of the skin.

## Main

Intracutaneously implanted electrochemical microneedles on wearable patches, such as the Libre Style (Abbott) and Dexcom G6/G7 (Dexcom), have advanced glucose measurements in diabetes management beyond painful finger pricks^1–6^, but these tools assess the levels of glucose diluted in the interstitial fluid (ISF), not the glucose concentration in blood. ISF glucose diffuses from blood capillaries^7–9^ in a delayed fashion and is present at lower concentrations than the clinically relevant glucose in circulation. Moreover, pH values and variations in ISF volume due to hydration level or temperature could challenge the accuracy of glucose determination. Importantly, the invasive nature of microneedle electrodes carries the risk for skin irritation and microbial infections. In response, several technologies have been considered for NIGM, to avoid the use of microneedles^10–12^. Terahertz (THz) spectroscopy detects glucose on the basis of its absorption spectrum at the 0.1–2.5 THz range^1–4,13^, but the low signal-to-noise ratio, broad absorption bands and overlapping spectra of glucose with other biomolecules challenge its sensitivity and specificity^14^. In the broad range of optical methods^3,14–16^, NIGM based on Raman scattering spectroscopy^17^ resolves specific vibrational spectral signatures of glucose at the fingerprint region of carbohydrates (900–13,000 cm^−1^)^18,19^. Although the method notoriously suffers from weak signals, signal-enhancing methods such as coherent anti-stokes Raman spectroscopy (CARS) and stimulated Raman scattering (SRS) could improve detection sensitivity^20–22^. Mid-infrared (mid-IR) absorption spectroscopy using optical, optoacoustic or thermal detection has also been explored for taking glucose measurements^23–30^, but its in vivo application has been challenged by non-glucose-specific absorption of light at superficial skin layers^9^.

To combine the strong signals produced by mid-IR absorption while overcoming the limitations of bulk glucose measurements in ISF, we developed DIROS, which operates by time-gating optoacoustic signals generated by mid-IR excitation^31^. We hypothesized that depth-gating would improve the sensitivity and accuracy of glucose sensing, on the basis of two key premises.

First, it can preferentially detect glucose in skin areas that are rich in microvasculature, i.e. in regions with high blood concentration. In-blood sensing can provide real-time reporting of glucose fluctuations, unlike measurements of ISF glucose, which are subject to delays. Moreover, the concentration of glucose in blood is the clinically relevant parameter, and it is higher than the ISF concentration, possibly leading to higher detection sensitivity. Second, it can minimize contributions from the metabolically inactive stratum corneum and from the overall epidermis; changes in skin humidity, superficial lipids and other molecules can contaminate glucose measurements and affect their reliability and reproducibility^29,30^.

Of critical importance in examining these two hypotheses was the depth probed by mid-IR excitation and optoacoustic detection, especially concerning its ability to reach subcutaneous, microvasculature-rich volumes. For this reason, we examined the depth achieved by mid-IR optoacoustics in vivo, using a broadband ultrasound detector (bandwidth, ~6–36 MHz; central frequency, ~21 MHz) to achieve an axial resolution of 30 µm at the upper frequency band (~45-µm resolution when all frequencies are used). Mid-IR measurements were contrasted with congruent microvasculature-sensitive optoacoustic measurements at 532 nm illumination, the latter serving as validation of the depths and structures probed. Then, using the merits of depth-selective optoacoustic detection, we examined the effects of signal localization in blood-rich volumes, validating the hypotheses above. In the following, we present the results of the depth interrogation and the DIROS glucose detection and discuss how DIROS may offer a preferred technology towards non-invasive glucose monitoring for improving diabetes management.

**Fig. 1:**
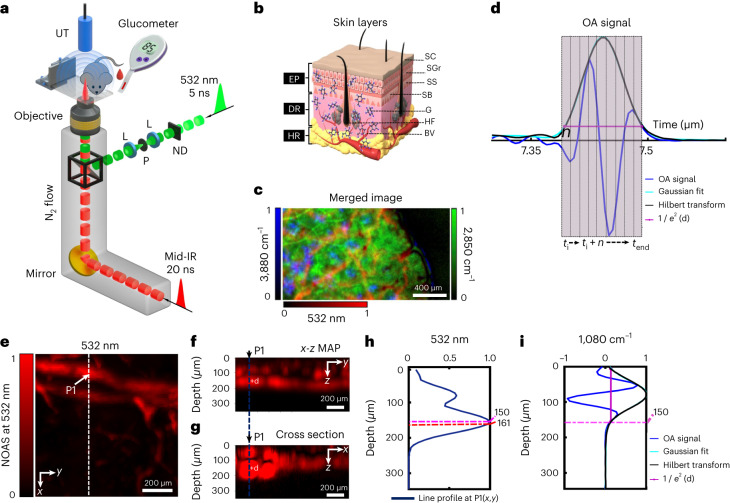
Imaging-depth capabilities of DIROS for label-free biomolecular sensing. **a**, Schematic of the combined mid-IR–visible (mid-IR/VIS) in vivo optoacoustic (OA) microscopy system for image-guided, non-invasive glucose monitoring (ND, neutral density filter; L, lens; P, pinhole). **b**, Schematic of different layers of mouse skin (EP, epidermis; DR, dermis; HR, hypodermis; SC, stratum corneum; SGr, stratum granulosum; SS, stratum spinosum; SB, stratum basale; SG, sebaceous glands; HF, hair follicles; BV, blood vessels; G, glucose). **c**, Merged mid-IR/VIS OA images; a representative example from ten independent experiments. **d**, Raw OA signal corresponding to a glucose-relevant wavenumber (1,080 cm^−1^; (d) indicates depth, *t*_i_ indicates initial gate time, *t*_end_ indicates final gate time), showing its Hilbert transform and the corresponding Gaussian fit. **e**, Representative maximum amplitude projected image (MAP) of a mouse ear at a 532-nm wavelength (NOAS, normalized OA signal) (*n* = 10). **f**, *x*–*z* MAP image, of the same micrograph in **e**, showing OA contrast distribution in depth. **g**, *x*–*z* cross-section image along the dashed line in **e**. In **f** and **g**, +d indicates the 1 / *e*^2^ depth (at 150 µm) defined by the Hilbert transform of the OA transient at a wavenumber of 1,080 cm^−1^. **h**, Depth contrast profile (532-nm wavelength) of a blood vessel at P1, marked by the dashed line in **f** and **g**. The depth of the maximum blood vessel contrast is indicated by the red dashed line (161 µm). **i**, Depth contrast profile at P1 for a wavenumber of 1,080 cm^−1^; the OA transient is also shown for reference (blue line). In **h** and **i**, the dashed magenta line indicates the penetration depth, defined by the 1 / *e*^2^ intensity of the Hilbert transform at a wavenumber of 1,080 cm^−1^ (150 µm). Data in **c**–**i** are representative of ten independent experiments (*n* = 10).

DIROS was implemented using an optical path shared by mid-IR and 532-nm-wavelength illumination so that mid-IR measurements could be cross-referenced with vascular features detected in the visible spectrum. The optical path (Fig. 1a) consisted of a pulsed mid-IR beam (pulse duration, 20 ns; 909–2,941 cm^−1^ and 3.4-11 μm spectral range) and a co-aligned 532-nm-wavelength pulsed beam (pulse duration, 3 ns). Both beams were focused on the surface of tissue (mouse ear) by a broadband reflective objective (see Methods for details). Optoacoustic measurements were collected in vivo using a focused ultrasound transducer placed on the opposite side of the tissue, establishing a slab geometry (Supplementary Fig. 1). For referencing purposes, we raster-scanned the sample under the sensor and generated merged mid-IR–visible optoacoustic images of tissue (Fig. 1c) to obtain anatomical references (Methods). Depth selection was implemented by gating the time-dependent optoacoustic signal (Fig. 1d) to select those generated within specific epidermal layers (Fig. 1b). Optoacoustic signals were processed using the Hilbert transform to ensure that the results correspond to the energy of the measured signal.

We previously postulated that mid-IR optoacoustic sensing can penetrate deeper in tissues than can conventional mid-IR optical techniques because it uses ultrasound and not optical detection, that is, it operates under strong optical attenuation only on the incident, but not the collection, path^31^. To experimentally examine the maximum depth reached, we obtained three-dimensional microvasculature maps from 1 × 1 mm^2^ scans from mouse ears in vivo, using 532-nm-wavelength excitation (Fig. 1e–g). Maximum amplitude projection (MAP) along the three dimensions enabled observation of vascular-rich volumes, as exemplified in the images. Then, we plotted the signal profiles collected at wavelengths of 532 nm (Fig. 1h) and 9,259 nm (wavenumber, 1,080 cm^−1^; Fig. 1i), taken from a volume with strong vasculature (point 1 (P1): see Fig. 1e–g), to assess the DIROS penetration depth—9,259 nm (wavenumber, 1,080 cm^−1^) was selected as a representative wavelength for glucose sensing (see Fig. 4h). The maximum depth reached was determined as the width of 1 / *e*^2^ of a Gaussian curve fitted to the Hilbert transform of the mid-IR optoacoustic signals (here, *e* is the exponential constant with a value of ~2.71828) (Methods). Figure 1i shows a representative example in which a penetration depth of ∼150 µm at a wavenumber of 1,080 cm^−1^ was reached, and the average maximum depth penetration at 1,080 cm^−1^ for all mice was ~140 µm (see Supplementary Fig. 2). Such depths are sufficient to reach capillary-rich layers at the epidermal–dermal junction of the human skin^32,33^ (Supplementary Fig. 3), so DIROS can be used to take measurements in people.

To further support these findings, we plotted the penetration depth of 4,900 optoacoustic measurement points, obtained by using DIROS to scan a 70 × 70 point grid. The measurements were collected from adjacent positions on the mouse skin (see Supplementary Fig. 4) and showed that depth variations at different skin locations are small. For instance, the relative s.d. (RSD) of the mean penetration depth at wavenumbers of 1,080 cm^−1^ and 1,034 cm^−1^ was 4% and 6%, respectively.

**Fig. 2:**
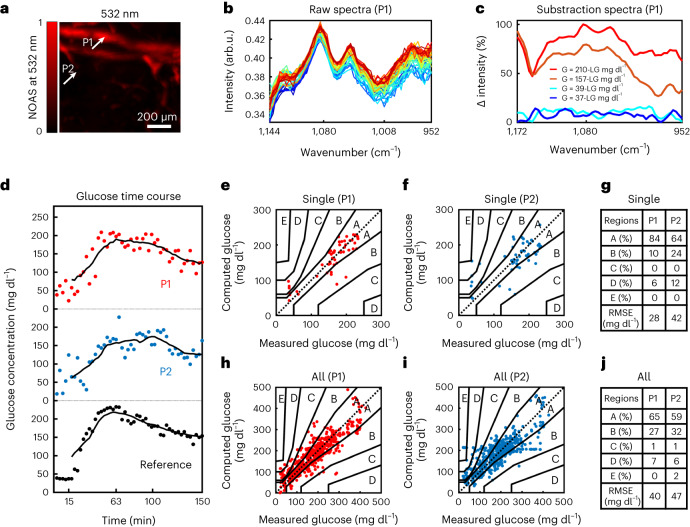
Location-selective non-invasive glucose monitoring with DIROS. **a**, OA image of a mouse ear (532-nm wavelength), representative of ten independent experiments (*n* = 10). **b**, Raw DIROS spectra at different glucose values over time, measured at P1. arb.u., arbitrary units. **c**, A plot of four DIROS spectra: two corresponding to the lower glucometer value following glucose administration, that is at 37 and 39 mg dl^–1^, and two corresponding to the highest observed values, that is at 157 and 210 mg dl^–1^. LG indicates “low glucose” with a value of 39 mg dl^–^^1^. **d**, Time profile of calculated glucose concentrations at P1 and P2 compared with reference blood glucose measurements. **e**,**f**, PCEGs showing the correlation between reference and OA glucose values at P1 (**e**) and P2 (**f**) from the mouse in **a**. The PCEG is divided into five regions (A–E) representing risk zones when determining glucose concentration; while zone A represents no risk, values falling in zone E could have dangerous consequences. **g**, Tabulation of the distribution of results per region, and RMSECVs by cross-validation, for **e** and **f**. **h**–**j**, PCEGs and tabulations for measurements at P1 and P2 for all ten mice in the study. Source data

Having verified that depth-dependent detection from capillary-rich layers is feasible, we investigated the glucose-detection performance of DIROS in blood and in ISF. To achieve this, we performed a glucose tolerance test in ten mice, in which a 20% glucose solution (2 g kg^-1^ body weight) injected into the abdomen of each mouse. First, for each mouse in the study, an absorption map at a wavelength of 532 nm was acquired using scanning steps of ~5 µm, to provide a morphological reference of the microvascular distribution in the area under the sensor and to select locations to test blood versus ISF measurements. To exemplify performance, we showcase results obtained from the same volume (Fig. 2a) used for depth evaluation in Fig. 1. All DIROS scans were performed at two points on the 532-nm maps: a first point (P1) at an area with vasculature presence, and a second point (P2) at an area with poor vascularization (that is, an area representative of measurement in the ISF). Ten baseline spectra (wavenumber, 900–1,300 cm^−1^) were recorded over the 10 min before glucose administration, and 90 spectra were recorded over the 150 min after glucose administration; the sensor was continuously alternated over the positions P1 and P2. Each spectrum was generated by averaging 1,000 optoacoustic signals. Each point in the spectrum corresponds to the peak amplitude value of the Hilbert transform of the averaged optoacoustic signal for the selected time gate. Each spectrum consists of measurements at 100 wavenumbers acquired in the 900–1,300 cm^−1^ region with a spectral step size of 4 cm^−1^, requiring 1.5 min for acquisition. After one spectrum measurement was completed from P1 or P2, the sensor was moved to the other position. For validation purposes, 0.6 µl of blood was obtained from each mouse every 3 min during the period in which a motorized stage moved the sensor from P1 to P2. The blood sample was analysed using a standard glucometer (Methods). Figure 2b shows raw optoacoustic spectra obtained from P1 at different time points, corresponding to different blood glucose concentration values (for colour coding, see Supplementary Fig. 5). Observation of the spectra revealed that optoacoustic intensity changed as a function of glucose concentration (which was found to be linear, as shown in Fig. 4). To illustrate the spectral change as a function of glucose concentration, we subtracted one baseline spectrum, obtained before glucose administration, from four spectra obtained after glucose administration (Fig. 2c): that is, two spectra corresponding to the lowest glucose concentrations (37 and 39 mg dl^–1^) and two spectra corresponding to the highest concentrations (157 and 210 mg dl^–1^) recorded by the glucometer. These four spectra show that, in all cases, there is a clear difference between the spectra and the baseline, and that the signal is well above the noise level. The change in intensity observed for the low glucose values is ~20% of the maximum change in intensity observed in the collected data set, confirming that the signal-to-noise ratio (SNR) is sufficient for in vivo glucose detection at physiological concentrations. The difference spectra of the raw spectra displayed in Figure 2b are shown in detail in Supplementary Figure 5.

To better understand the nature of the collected spectra, we performed Fourier transform infrared (FTIR) spectroscopy measurements on phantoms (namely, liquid solutions of biomolecules found in tissues) mimicking the composition of blood and skin (as above), including key components such as cholesterol, triglycerides and keratin. The FTIR experiments indicate potential interference and overlap from the measured metabolites only at concentrations considerably above the physiological range (Supplementary Fig. 6a). Lactate showed the highest potential interference, with two distinct peaks partially overlapping with those from glucose (wavenumbers, 1,040 and 1,125 cm^−1^). For this reason, we further performed DIROS measurements of a solution containing lactate and albumin at physiological concentrations (6–12 mg dl^–1^ and 2,000–4,500 mg dl^–1^, respectively)^34,35^, and observed that lactate did not contribute to a spectrum that interferes with the glucose spectrum, possibly owing to its low concentration (see Supplementary Fig. 6b). Fetal bovine serum (FBS) may also interfere with the measurements; however, it exhibits a slowly varying spectral component and no distinct peaks in the spectral region of interest that could interfere with the glucose measurements. Therefore, similar to water contributions, it can be accounted for as a background contribution in the multivariate analysis (MVA) used to determine glucose levels, described in the following text.

To quantitatively investigate the relation between spectral changes and blood glucose concentration beyond the observation of raw spectra, we used a MVA method based on partial least squares regression (PLSR). MVA is the typical approach for computing analyte concentrations from spectroscopic glucose sensors^36^, and it considers the structure of the entire spectrum (100 variables) when computing a single glucose value in the presence of other contributors (metabolites) in tissue. Given a number of spectra (measurements) and ground-truth glucose values (obtained from the glucometer), the PLSR describes the spectral data as a linear combination of a new set of spectral components (basis spectra) and identifies the subset of components that provides the most information about the glucose level. Then, it computes a glucose value on the basis of the particular combination of these spectral components that describes a given spectrum. We applied a leave-one-out cross-correlation, whereby each spectrum used for a glucose measurement was excluded once from the decomposition to basis spectra (Methods) to determine features that represent spectral variation.

Using MVA analysis, we plotted the glucose values obtained from P1 and P2 versus the glucometer values over the time course of a measurement (Fig. 2d). Although both P1 and P2 exhibited changes in glucose levels corresponding to the administration of glucose, the data from location P1 more closely resembled the blood glucose dynamics recorded by the glucometer. The curves also showed a delayed appearance of glucose measured at position P2. This is consistent with the fact that changes to glucose levels in interstitial fluids, represented in our work by measurements at P2, appear in a delayed manner compared with the dynamics of blood glucose, represented by measurements at P1. We also performed time-course control experiments in which phosphate-buffered saline (PBS) was injected in three mice. The results showed a minimum baseline increase that was essentially constant throughout the time course of measurement (Supplementary Fig. 7), supporting the interpretation that the signals in Figure 2d are due to glucose injection.

To study the accuracy of the glucose measurement in relation to the two measurement locations, we applied the Parkes consensus error grid (PCEG) to locations P1 and P2, for the mouse shown in Figure 2a (Fig. 2e–g) and for the entire data set collected from all mice (Fig. 2h–j). The PCEG is divided into five regions (A–E), representing degrees of accuracy of glucose estimations. Values falling into different zones have various levels of accuracy: those in zone A are the most accurate (within 20% of the reference measurement), and those in zones D and E represent erroneous readings^37^. Visually, the measurements at P1 appear less scattered and better confined to zone A than do the measurements at P2. In accordance, the root mean square error cross-validation (RMSECV) value between the measurements at P1 and P2 and the reference glucometer values were found to be 28 mg dl^–1^ versus 42 mg dl^–1^ for the example mouse in Figure 2a, and 40 mg dl^–1^ versus. 47 mg dl^–1^ for the entire cohort.

The glucose-measurement results shown in Figure 2 were obtained by selecting areas with and without vasculature, but without depth selection, confirming the hypothesis that measurements from blood-rich volumes are more accurate than are measurements in ISF. The next critical step, and a key point of the development of the DIROS sensor, was to examine whether depth selection could further improve DIROS performance beyond the capabilities of current sensors. Time gating avoids bulk measurements and can localize readings from blood-rich layers or volumes that lie under the epidermis. Therefore, this approach does not integrate non-specific contributions from the epidermis and bulk ISF measurements, offering measurements that can be labelled as in-blood. Although our work is guided by images, it can be applied in vivo without imaging by targeting the epidermal–dermal junction layer, which is rich in blood-filled capillaries across the skin in animals and humans (Supplementary Fig. 3).

**Fig. 3:**
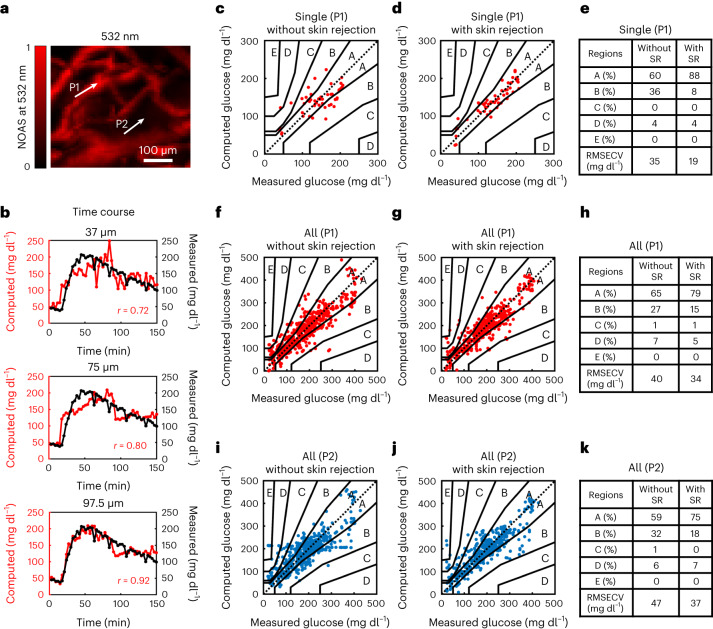
Depth-selective non-invasive glucose monitoring with time-gated OA sensing. **a**, OA micrograph (532-nm wavelength), representative of ten independent experiments (*n* = 10). **b**, Time course of glucose measurements at different depths (37, 75 and 97.5 μm) at P1, compared with reference blood glucose values. **c**,**d**, PCEGs for a representative experiment in a single mouse. Measurements were taken at P1 without skin rejection (SR) (**c**) and with SR (**d**). **e**, Table comparing the distribution of results per region, and average RMSECV with and without SR at P1. **f**,**g**, PCEGs for ten experiments. Measurements were taken at P1 without SR (**f**) and with SR (**g**). **h**, Table comparing the distribution of results per region, and average RMSECV for measurements with and without SR at P1. **i**,**j**, PCEGs for ten experiments. Measurements were taken at P1 without SR (**i**) and with SR (**j**). **k**, Table comparing the distribution of results per region, and average RMSECV for measurements with and without SR at P2. Source data

Glucose concentrations were computed with and without gate selection at points P1 and P2. Showcased results (Fig. 3) are from a different mouse (Fig. 3a) than the one shown in Figures 1 and 2, to illustrate the diversity seen in the collected vascular maps. Similar to the analysis in Figure 2, we selected two measurement locations: one with higher (P1) and one with lower (P2) microvascular density. However, here we applied a time-gate algorithm (Methods) that was optimized so that the optoacoustic signal could be sectioned to obtain spectra at time gates (depths) that minimized the error between the DIROS measurements (that is, the Hilbert transform of the optoacoustic signal) and the reference glucose measurements. Different layers correlated differently with the measured glucose values, confirming that DIROS performance varies with depth. The layer at a depth of 97.5 ± 20 µm gave an optimal error minimization for all mice and was therefore selected as the gate for all mice in all measurements. Insight into the effects of time gating is seen in Figure 3b, which compares glucose values at different gates, that is, at different skin layers (depths), with the reference measurements and shows that the selected time gate provides the best match. Superficial measurements can correspond to bulk measurements from the stratum corneum and top of the epidermis, similar to measurements performed by other sensors, and show a worse match to the glucometer values, offering a first validation of the main DIROS hypothesis that depth selection can improve accuracy. We computed the Pearson correlation coefficient between DIROS measurements and glucometer values to quantify the match between the two techniques. We found a Pearson correlation coefficient of *r* = 0.92 for measurements at a depth of 97.5 µm, but lower correlation coefficients of *r* = 0. 80 and *r* = 0.72 as the gate was moved towards the skin surface.

To further validate the effect of depth selection, we plotted the PCEG with and without gate selection (Fig. 3c–k) and show an up to approximately twofold improvement in sensitivity when using the optimal gate (Fig. 3e). The representative results from a single mouse show that measurements from microvascular-rich volumes with depth selectivity by rejecting signals generated by the epidermis (i.e. skin rejection), (Fig. 3d) yielded higher accuracy (88% of the points in zone A) than do measurements obtained without skin rejection (with only 60% of the points in zone A, see Fig. 3c,e). When comparing the results from all mice, 79% of the measurement points fell in zone A of the PCEG for the P1 location using skin rejection, whereas only 65% of the measurement points fell in zone A without the time gate (Fig. 3f–h). Therefore, the most sensitive performance was achieved for measurements obtained from the P1 position after applying a time gate. Overall, the root mean squared errors (RMSEs) for the entire cohort of mice improved from 47 mg dl^–1^ for bulk ISF measurements (P2; Fig. 3k) to 34 mg dl^–1^ for measurements of blood-rich volumes with depth selection (P1; Fig. 3h).

**Fig. 4:**
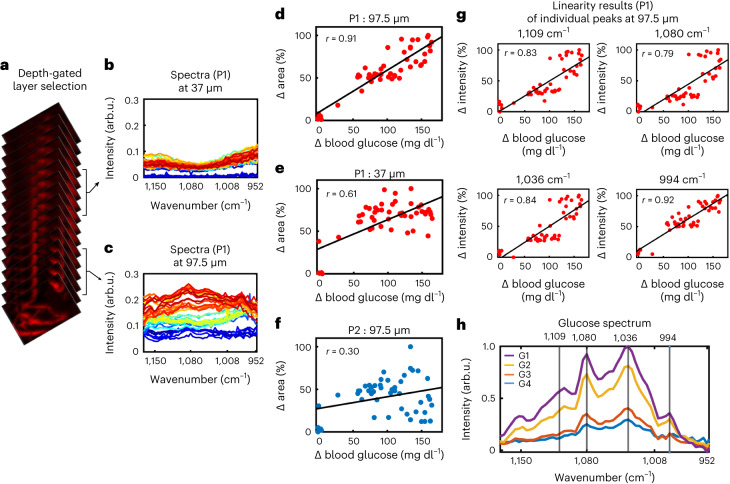
Different time gates measure different spectral compositions at different skin layers. **a**–**c**, A representation of different section slices (**a**), selected by time-gating the OA signals and corresponding spectra for P1 at depths of 37 µm (**b**) and 97.5 µm (**c**). **d**–**f**, Plots of the change of the area under the curve of the subtracted spectra as a function of reference glucose values determined by the glucometer for the 97.5-µm-deep layer at P1 (**d**) the 37-µm-deep layer at P1 (**e**) and the 97.5-µm-deep layer at P2 (**f**). **g**, Correlation of intensity changes as a function of reference glucose values determined by the glucometer for peaks in the spectrum, corresponding to the 1,109, 1,080, 1,036 and 994 cm^−1^ wavenumbers. **h**, Glucose spectra measured by DIROS in water solution at different concentrations (G1, 2,500 mg dl^–1^; G2, 1,250 mg dl^–1^; G3, 625 mg dl^–1^; G4, 312 mg dl^–1^). Source data

To better elucidate the differences in glucose measurements at different time gates (Fig. 4a), we plotted the spectra collected from a superficial layer (at 37 µm, Fig. 4b) and a deeper layer (at 97.5 µm, Fig. 4c) from location P1 at different time points, that is, different glucose concentrations. The spectra recorded from the deeper layer show increasing intensities as glucose concentrations increase (for colour coding, see Supplementary Fig. 5). Furthermore, it is visually evident that the changes in the deeper layer are more prominent than in the superficial layer. Moreover, in contrast to the spectral changes observable at the superficial layers, which resemble the spectrum of water, the spectra at the deeper layers resemble that of glucose (see Fig. 4h).

To study the linearity of the scaling of the spectra observed, we plotted the area under the curve versus glucose concentration for the deep and superficial layers at position P1 (Fig. 4d,e) and for the deep layer at position P2 (Fig. 4f). We observed approximate linear correlations at all locations; however, the best correlation (*r* = 0.91) was obtained for the deeper layer at position P1, which is closer to vasculature and rejects signal contributions from the skin. Measurements at the more superficial layer at position P1 gave a correlation coefficient of *r* = 0.61, whereas measurements from the deeper layer at the poorly vascularized position P2 exhibited the worst correlation (*r* = 0.30).

Although area-under-the-curve plots are useful in understanding the energy signal of the entire measurement, we were also interested in investigating whether individual wavenumbers would be sufficient for glucose prediction. Therefore, we plotted the intensities of four wavenumbers corresponding to peaks in the glucose spectrum (Fig. 4h), obtained from the 97.5-µm-deep layer of P1, as a function of glucose concentration (Fig. 4g). Individual wavenumbers also showed good correlation with the measured glucose values, with the peak at 994 cm^−1^ demonstrating the highest correlation at *r* = 0.92. A possible reason for a better performance at 994 cm^−1^ than, for instance, at 1,080 cm^−1^, is that 994 cm^−1^ has lower interference from spectral contributions of other tissue chromophores than do other glucose peaks (Supplementary Figs. 6 and 8).

We demonstrated depth-selective glucose sensing in vivo, capable of reaching micro-vessels at depths of >100 µm and therefore enabling measurements from volumes with high blood concentration. Furthermore, by rejecting signals from superficial skin layers, DIROS minimizes the sensitivity to non-glucose-specific signals from the epithelium that are known to contribute to a highly heterogeneous skin appearance when observed in the mid-IR range and render optical NIGM measurements unreliable^26,29,30^. We observed that in blood measurements, that is, measurements from capillary-rich volumes, offered higher sensitivity and better precision in recording the dynamics of blood glucose variation than do ISF measurements. Likewise, using time-gated detection to reject signals from the skin surface improved the glucose measurement accuracy over bulk measurements.

We presented observations from both raw data and multivariate data-analysis methods. Inspection of raw data demonstrated that even individual spectral points can report on glucose concentration, as further detailed in Figure 4. Similar analysis was performed with Raman spectra^19^, allowing a preliminary glimpse into the relative sensitivity between Raman and DIROS. Raman spectra demonstrated observable spectral differences for glucose concentrations in the 256–456 mg dl^–1^ range, whereas DIROS raw spectral analysis could detect glucose concentration changes in the range below 100 mg dl^–1^.

Our study has certain limitations. The measurements were obtained from mice and not humans, owing to what appears to be an erroneous interpretation of the European Union Medical Device Regulation 2017/745, which came into effect in May 2021. Although regulation 2017/745 is aimed at commercial developments regarding the placement of medical products in the market or in service for patients, German authorities interpret this regulation as also applying to research investigations, making research challenging and requiring approvals similar to those required for commercial systems. Nevertheless, the epidermal–dermal junction has been found at virtually identical depths of ~20 µm in nude, white (CD-1) and grey (C57BI/6) mice^38^ (see also Supplementary Fig. 3b for different skin locations in mice); the epidermal–dermal junction depth in humans ranges from 20 to 80 µm in many skin locations^39,40^. Because DIROS can be used at depths of up to 100 µm, it is plausible that the results demonstrated herein will be confirmed not only in other mice, but also in people once DIROS receives approval for human studies. Although DIROS measurements might be affected by sweat, we anticipate that DIROS will be applied to dry skin and/or anhidrotic skin locations, for example the lip, earlobe or nail-fold; however, the study of the influence of sweat should be also considered in the future, under controlled protocols, so that the sensor can be used in more locations. A second limitation was that sensor signals might contain fluctuations owing to laser instability and electronic noise. We found that signal fluctuations had a mean of 1.4% of the maximum observed signal (Supplementary Fig. 9). We partially compensated for this instability by using a high number of averages and collected spectral points. In the future, a reference optoacoustic arm could account for such fluctuations, leading to a reduction in the number of acquisition points required for averaging, thus accelerating the measurement process.

DIROS could be extended beyond glucose measurements to other metabolites, such as lactate and lipids. This could allow, for instance, the development of a continuous metabolic sensing system to alert a user to deviations from healthy metabolic parameters. In summary, the method presented here is a powerful new tool for precise determination of clinically relevant blood glucose levels that could pave the way for significant advances in diabetes management.

## Methods

### Combined visible and mid-infrared optoacoustic microscopy

A pulsed quantum cascade laser (QCL) (MIRcat, Daylight Solutions), with a tuning range from 3.4 µm to 11 µm, 20-ns duration and a repetition rate of 100 kHz, was used as the optoacoustic excitation source. Additionally, a 3-ns laser beam at 532 nm (Cobolt, Hübner Photonics) was integrated with a flip-mirror sharing the same optical path of the QCL (Fig. 1a). Both visible- and mid-IR-output laser beams were focused to the sample by a ×36 reflective objective (Newport Corporation). Optoacoustic signals from the sample were detected with an ultrasonic transducer with a central frequency of 21 MHz (Imasonic). To evaluate the co-registration accuracy between the two systems, we obtained carbon-tape images at 532 nm, the wavelength used to enable visualization of hemoglobin-based contrast, and at three specific wavenumbers in the mid-IR range, corresponding to glucose, lipid and protein detection in the skin (1,085, 2,850 and 1,587 cm^−1^, respectively; Supplementary Fig. 10a-c). Comparison of the line profiles through the image centre along the *x* and *y* axes (Supplementary Fig. 10d) showed excellent agreement between all images (Supplementary Fig. 10e–f). The merged visible and mid-IR optoacoustic image (Supplementary Fig. 10i) revealed slight differences in the spatial localization between the two images, calculated by using 100-µm line profiles along the *x* and *y* axes (Supplementary Fig. 10j–k). This slight difference was taken as a reference when selecting the blood vessels, and because the vessel diameter for selective localization of glucose monitoring was greater than 10 µm, the selective localization was confined inside the vessels.

### Glucose tolerance tests and in vivo mid-infrared optoacoustic spectroscopy

For location-selective non-invasive glucose monitoring in vivo, we first used the visible laser integrated into our mid-infrared optoacousctic microscopy (MiROM) system to localize vascular-rich regions, and the image of a mouse ear was acquired using a wavelength of 532 nm. Images of mouse ear tissue were then acquired at a wavenumber of 2,850 cm^−1^ using MiROM to visualize skin heterogeneity (see Fig. 1c). The acquired signals at 532 nm and 2,850 cm^−1^ were averaged over 50 consecutive signal cycles. Using these images, we selected two locations (P1 and P2) to test the correlation between spectral changes (in the range from 900 cm^−1^ to 1,300 cm^−1^) and blood glucose concentration. To this end, glucose tolerance tests were performed in ten mice at P1 and P2. Five baseline spectra were simultaneously acquired over 10 min before glucose injection, and 45 spectra were collected for 150 min after glucose injection at each point. For each in vivo mid-IR spectra, we obtained a reference blood glucose value using a glucometer (Contour Next, Ascensia Diabetes Care) to correlate spectral changes and blood glucose concentration. For each glucose tolerance test, a total of 50 blood glucose reference values and 50 in vivo mid-IR optoacoustic spectra (per measurement point) were obtained.

### Multivariate analysis

The collected spectra were constructed by taking the maximum intensity of the Hilbert transform applied to the retrieved mid-IR optoacoustic transients. Principal component analysis was applied to the series of optoacoustic spectra collected for each glucose tolerance test (that is, 50 spectra per test for each point) to determine their common features. Because the size of the spectrum data set was smaller than the parameter of independent variables at the wavenumber, a PLSR algorithm and cross-validation were used to calculate the glucose concentration. The algorithm enabled the rotation of the coordinate system of the data space and the generation of new components, namely a latent variable. The algorithm thus maximizes variance and correlation between the variables coming from the measured spectrum data and reference glucose concentrations. The PLSR model was constructed after preprocessing through mean scale, and a leave-one-out cross-validation was performed for each glucose tolerance test to obtain the root mean square error of cross-validation (RMSECV). For the PLSR analysis, Matlab (Matlab 2019a) and PLS (PLS_Toolbox 8.9.2, Eigenvector Research) were employed.

### Maximum penetration depth of mid-IR optoacoustic signals

Optoacoustic sensing is a positive-contrast detection method, whereas conventional optical detection is a negative-contrast method. Therefore, optoacoustic sensing allows a higher signal-to-noise ratio (SNR) with depth than does conventional optics, enabling a higher proportion of the initial irradiation energy to be detected in the form of optoacoustic signals and allowing detection from deeper depths. In fact, even depths at which irradiation has dropped by up to 14% (1 / *e*^2^) of its initial value generate a detectable optoacoustic signal with DIROS. Comparatively, at depths at which irradiation has dropped by up to 37% (1 / *e*) of its initial value, signals can barely be detected using conventional optical detection. To calculate the penetration depth, the Beer–Lambert law, which states that penetration depth is inversely correlated to a sample’s optical absorption coefficient, is applied. Owing to the higher SNR, a larger width of 1 / *e*^2^ of Hilbert transform can be used to calculate the depth of DIROS. By contrast, a smaller width of 1 / *e* of Hilbert transform is used in conventional optical detection methods, owing to lower SNR.

### Skin-sectioning depth-selective glucose sensing

To avoid anatomical structures in skin areas with low glucose content for more precise in-blood glucose detection, a specific time window of optoacoustic signals was used in order to interrogate deeper vessels. The width of the time window (*w*) was selected to be 7.5 µm in the range of the width of 1 / *e*^2^ of the Hilbert transform of the optoacoustic transient, representing the achievable depth at the corresponding wavenumber for glucose detection of optoacoustic spectra at two locations (P1 and P2). For each *w*, the window was shifted in 7.5-µm steps, and for each position of the window, a spectrum was generated for the glucose tolerance test. The PLSR model was constructed for each spectrum acquired by time-gated signals, and a leave-one-out cross-validation was performed for the spectral information corresponding to certain depth layers. The RMSECV between the reference and the glucose values in the specific window was calculated. This process of providing spectral information along the skin depth was used as a skin-rejection window to calculate glucose concentrations only from deeper seated vessels.

### Sample preparation and experimental protocol for in vivo glucose monitoring

All mouse experiments were performed according to the guidelines of the committee on Animal Health Care of Upper Bavaria, Germany (approval number Az ROB-55.2-2532.Vet_02-14-203). The mice were maintained in an individually ventilated cage system (Tecniplast) at 22 °C ambient temperature, a relative humidity of ~50% and a regular 12-hour day–night cycle, in our specific-pathogen-free mouse facility at the Center for Translational Cancer Research of the Technical University of Munich. Mice aged 4–8 weeks were used in the study. Female athymic nude-Foxn1^nu^ mice (Envigo, Germany) were selected for the glucose tolerance tests. During all the measurements, the mice were anesthetized with 1.6% Isoflurane (CP-Pharma) and 81 pm oxygen as the carrier gas. The mouse heart rate, body temperature and the SpO_2_ were controlled by a monitoring device (Physio suite, Kent Scientific). The imaging of all the mice was performed on the left ear.

After acquiring the baseline data, 2 g kg^–1^ (body weight) glucose (Braun, 20% glucose) was injected into the mouse intraperitoneally. For reference glucose measurements, glucose in the blood was measured in parallel with glucometer measurements using test strips (Contour Next, Ascensia Diabetes Care). The blood was extracted from the caudal vein, and the mice were euthanized immediately after measurements.

### Reporting summary

Further information on research design is available in the Nature Portfolio Reporting Summary linked to this article.

## Supplementary information

Supplementary Information Supplementary Figs. 1–10.

Reporting Summary

## Source data

Source Data Fig. 2 Processed numerical data points of time course and consensus error grid analysis.

Source Data Fig. 3 Processed numerical data points of time course and consensus error grid analysis.

Source Data Fig. 4 Numerical data points of linearity results.

## Data Availability

The data that support the findings of this study are available from the corresponding authors upon reasonable request. Source data are provided with this paper.
