# Supplementary material for: Non-invasive measurements of blood glucose levels by time-gating mid-infrared optoacoustic signals
Source: Nat Metab. 2024 Mar 27;6(4):678–86. doi: 10.1038/s42255-024-01016-9 (PMC11052715; doi:10.1038/s42255-024-01016-9)
Supplement: Supplementary file 1 — Supplementary Figs. 1–10. [file 42255_2024_1016_MOESM1_ESM.pdf]

nature metabolism

Letter

https://doi.org/10.1038/s42255-024-01016-9

Non-invasive measurements of blood glucose levels by time-gating mid-infrared optoacoustic signals

In the format provided by the authors and unedited

Supplementary Figures

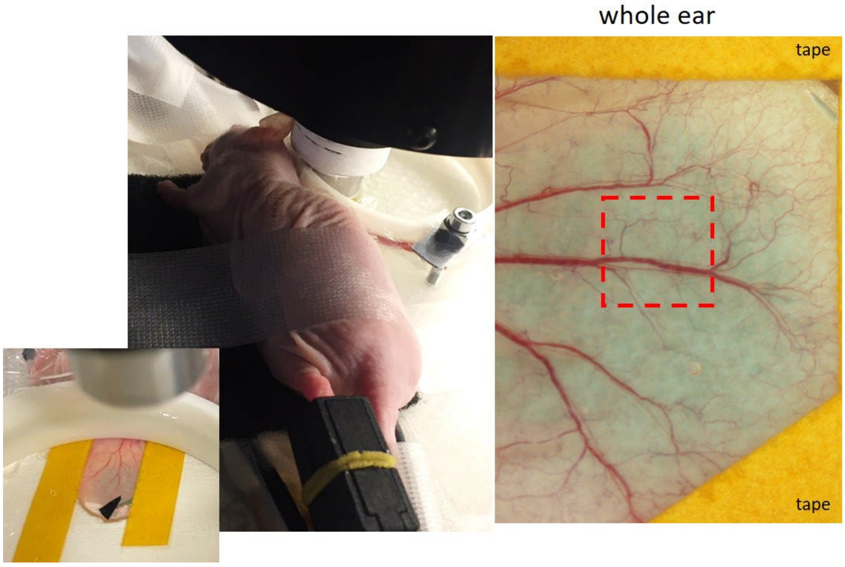

Supplementary Figure 1: Photograph of a mouse ear showing the scanning area. Photographs (from ten independent experiments) showing positioning of an anesthetized mice and mouse ear vasculature. A scanning area of 70x70 points, indicated by red-dashed line, was measured in all 10 mice.

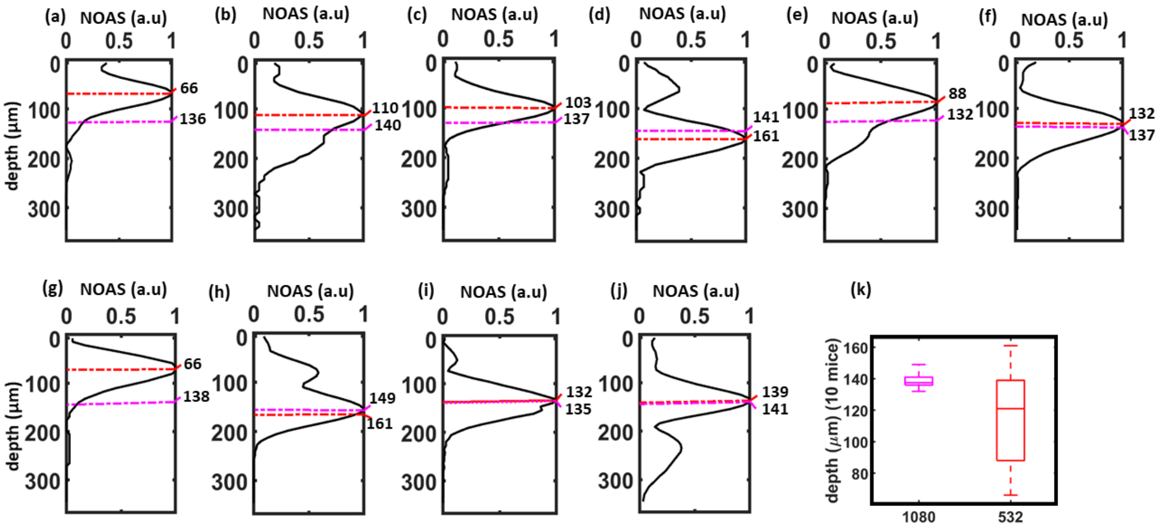

Supplementary Figure 2: Comparative DIROS/VIS-OA depth analysis at 1080 cm^{-1} and 532 nm for ten mice experiments. (a-j) Depth contrast profiles at P1 (blood vessel location) for 532 nm excitation. The penetration depth (1/e^2 of the Hilbert transform) at P1 for 1080 cm^{-1} and the depth for the maximum intensity obtained for blood vessels at 532 nm, are indicated by magenta and red dashed lines, respectively. (**k**) Comparison of the depth distribution of blood vessels (obtained at 532 nm) and the distribution of penetration depths reached by DIROS (at 1080 cm^{-1}), summary of 10 mice. NOAS = normalized optoacoustic signal; box plots indicate the upper and lower quartiles (box limits), median (center line) and minimum and maximum values (whiskers).

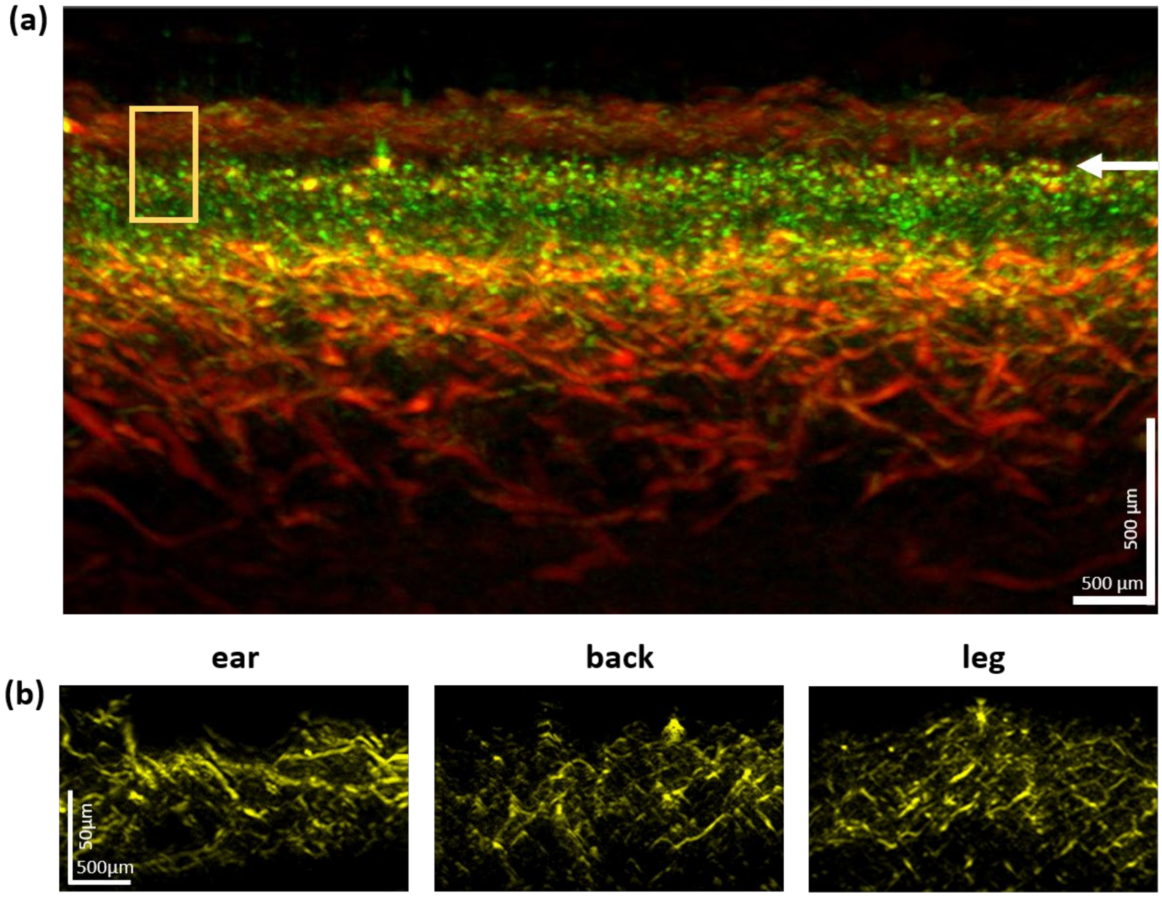

Supplementary Figure 3: Cross-sectional raster-scan optoacoustic mesoscopy images from human and mice skin. (a) Cross-sectional raster-scan optoacoustic mesoscopy (RSOM) image of human skin. The arrow points to the epidermal-dermal junction, which is a superficial layer rich in micro-vasculature. Green color marks the capillary loops in this layer. The yellow box indicates the depth scanned by DIROS, according as showcased in **Fig. 1**. Reproduced from Aguirre J., et al., Nature Biomedical Engineering (2017).[32] **(b)** Cross-sectional RSOM images of the mouse ear, back and leg. Representative example from 3 independent measurements (n=3). Similar microvasculature distribution is found at different mice skin locations.

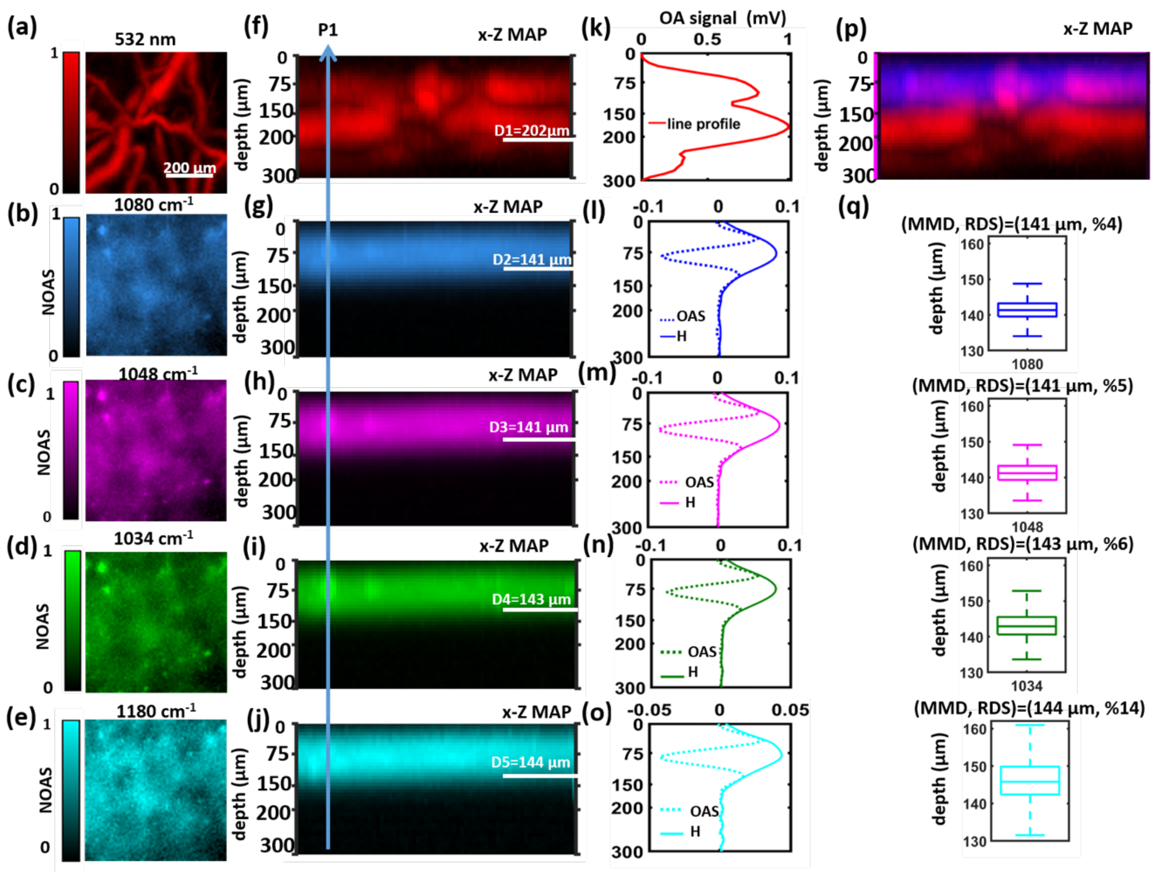

Supplementary Figure 4: Imaging depths achieved by DIROS in mouse skin *in vivo*. Representative xy-projected mouse ear images obtained from 3 independent experiments at **(a)** 532 nm, **(b)** 1080 cm^{-1}, **(c)** 1048 cm^{-1}, **(d)** 1034 cm^{-1}, and **(e)** 1180 cm^{-1}, **(f-j)** Maximum projections of corresponding xy images (**a-e**). (**k**) Line profile of blood vessels at P1 (blue line on panel **f**). **(l-o)** Depth profiles of mid-infrared (IR) optoacoustic (OA) transients with Hilbert transform at P1 (blue line on panels **g-j**). **(p)** Merged visible (**f**) and mid-IR (**g**) optoacoustic images. **(q)** Mean maximum depths achieved by DIROS at 1080 cm^{-1}, 1048 cm^{-1}, 1034 cm^{-1}, and 1180 cm^{-1} for 4900 measurement points on mouse skin; box plots of optoacoustic contrast for each channels in the micrographs (g-j) indicating the upper and lower quartiles (box limits), median (center line) and minimum and maximum values (whiskers). 3 biologically independent samples were used for the experiments (n = 3). All the data are presented as mean values +/- SEM. H – Hilbert transform, MMD – mean maximum depth, NOAS – normalized optoacoustic signals, OAS – Optoacoustic signal, SEM–standard error of mean.

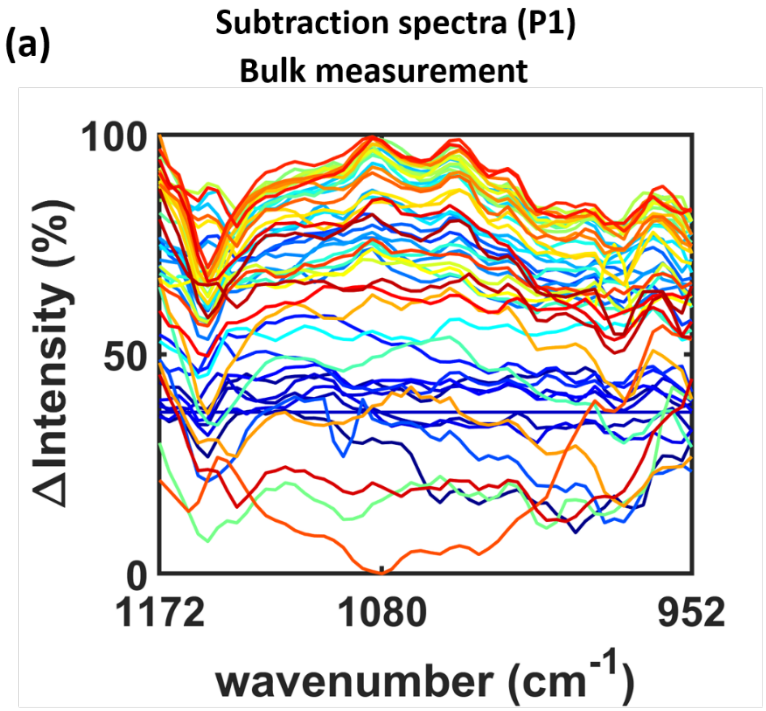

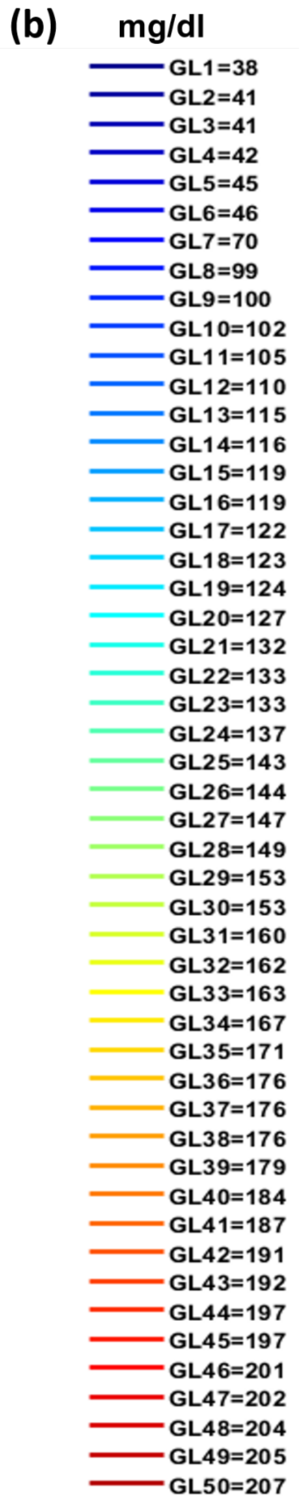

Supplementary Figure 5. Differential spectra analysis at time points post glucose administration. Difference spectra (**a**) generated as a subtraction of a baseline spectrum (obtained prior to glucose administration) from 50 spectra obtained at different time-points post glucose administration, and hence at different glucose values –color-coded in the legend in (**b**).

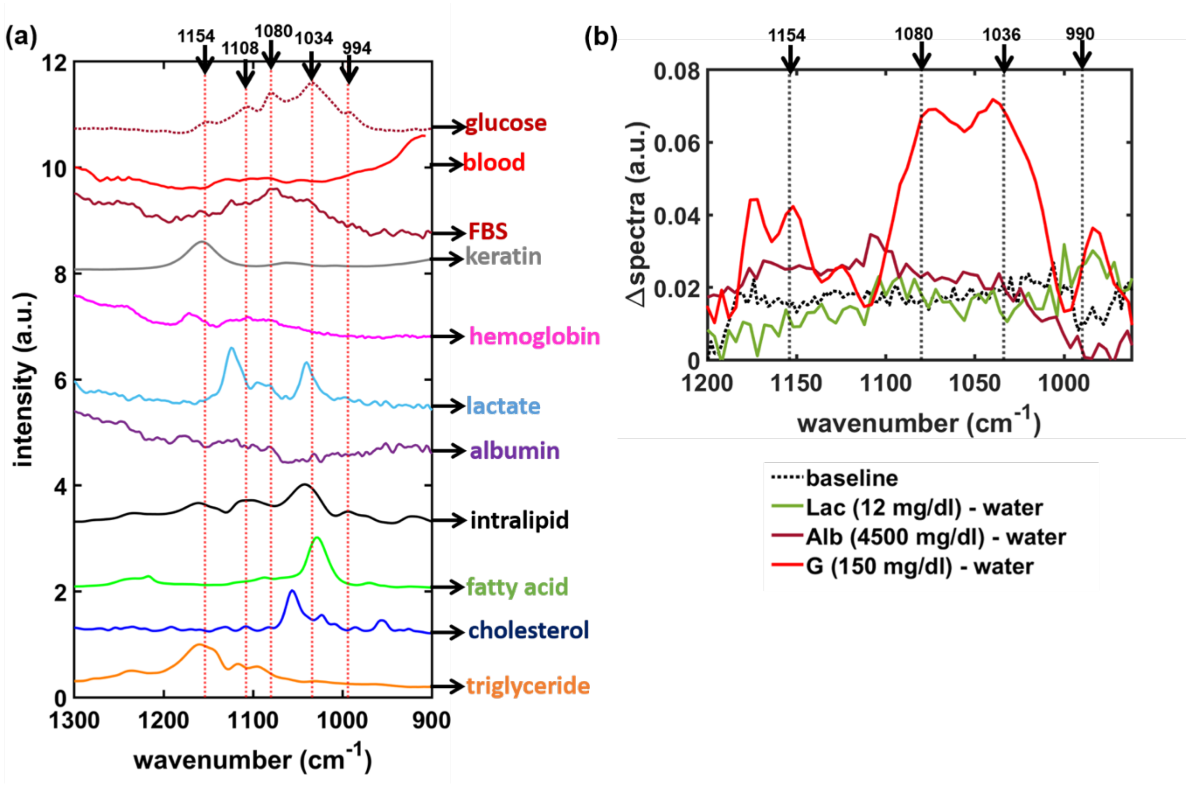

Supplementary Figure 6: **Comparison of spectral features of glucose and relevant components in tissue and blood by FTIR and DiROS spectroscopy. (a)** The five major absorption peaks of glucose are marked with vertical red dotted lines in the mid-infrared range (1300 to 900 cm^{-1}) for comparison with relevant blood and in tissue components at concentrations above the physiological range. A potential source of interference for glucose sensing is the two distinct peaks of lactate (1040 and 1125 cm^{-1}) that partially overlapping with those from glucose (1036 cm^{-1} and 1108-1154 cm^{-1}). **(b)** DIROS spectra of lactate, albumin, and glucose at physiological concentration after subtraction of water spectrum. The dashed-vertical lines indicated main absorption bands of glucose.

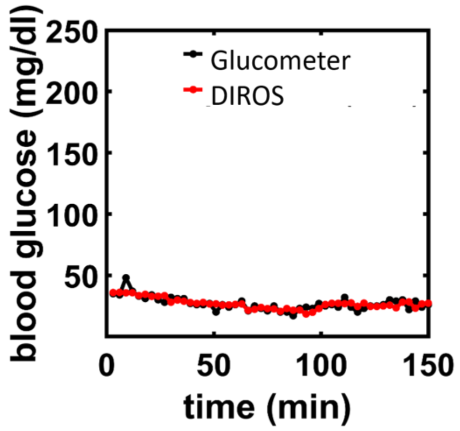

Supplementary Figure 7: Time course of glucometer and DIROS measurements after injection of phosphate buffer saline (PBS) in a mouse. When PBS in injected instead of glucose we observed a virtually constant OA response throughout the time course of the measurement, as compared to the reference blood glucose; further supporting that DiROS retrieve glucose-specific signals.

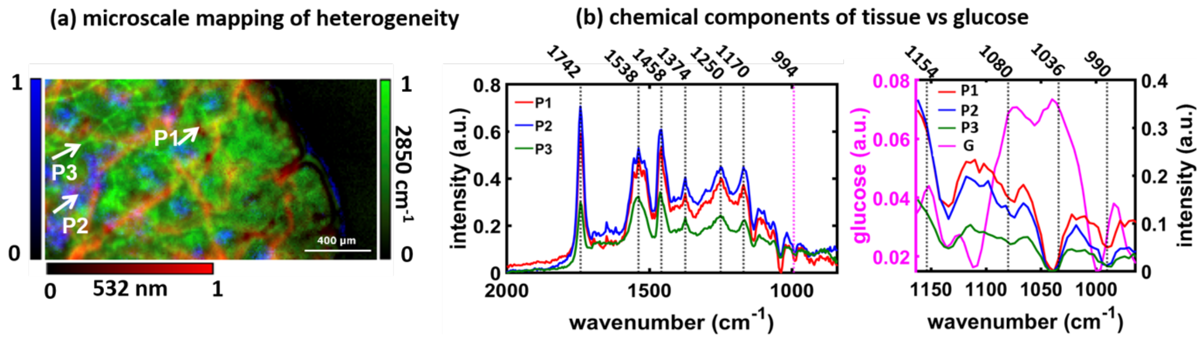

Supplementary Figure 8: The *in vivo* spectra of mouse skin obtained by DIROS. (a) Merged mid-IR/visible optoacoustic images. Three locations are marked: P1 - blood vessel, P2 - sebaceous gland, P3 – wrinkle. (**b**) Overlaid *in vivo* skin spectra of P1, P2, and P3 (indicated by arrows in (**a**)). The panel to the right shows glucose spectrum overlaid with the spectra of P1, P2, and P3 in the 964 - 1164 cm^{-1} range. G – glucose at 150 m/dl.

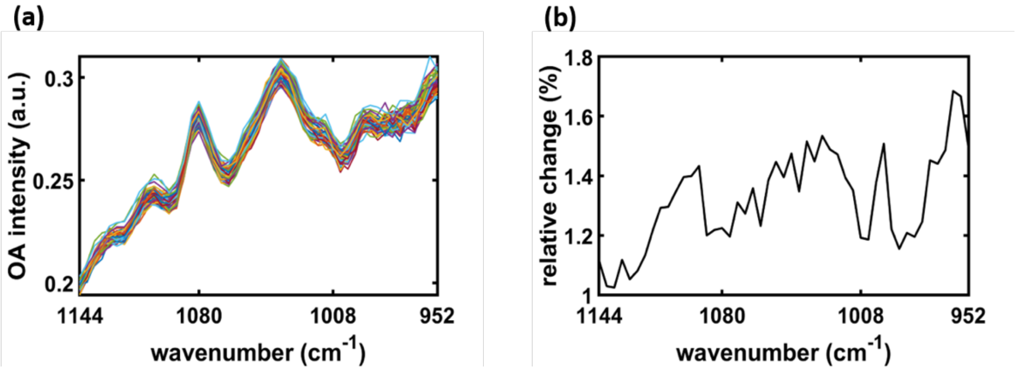

Supplementary Figure 9: System fluctuations in DIROS. (**a**) 48 representative spectra collected from repeated measurements of 5 g/dl glucose (in water), (**b**) Relative change (%) of the 48 spectra shown in (**a**). OA – optoacoustic. The maximum system fluctuation is about 1.7%, with a mean of 1.4%.

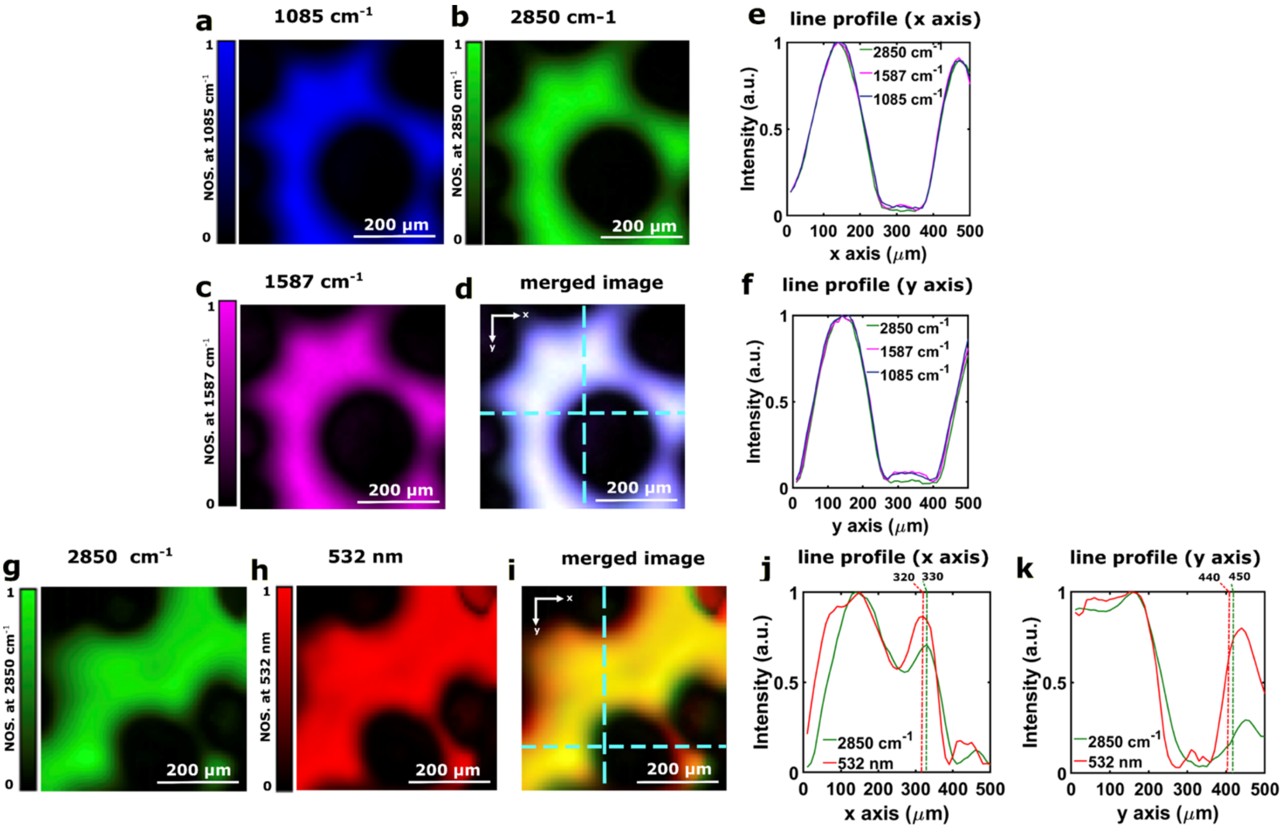

Supplementary Figure 10: Co-registration accuracy test for the combined VIS-OA/MiROM system. Micrograph of carbon tape at **(a)** 1085 cm^{-1}, **(b)** 2850 cm^{-1}, **(c)** 1587 cm^{-1}. **(d)** Merged image of (a-c). Intensity line profile at the dashed line in (d) along the **(e)** x-axis, **(f)** y-axis. Data in **a**–**d** are representative of three independent experiments. Micrograph of carbon tape at **(g)** 2850 cm^{-1} and **(h)** 532 nm. **(i)** Merged image of (g-h). Intensity line profiles at the dashed line in (**i**) along the **(j)** x-axis and **(k)** y-axis. While negligible misalignment between mid-IR micrographs was observed, a maximum deviation of 10 µm between visible and mid-IR micrographs was found. Data in **g–i** are representative of 3 independent experiments (n=3).
