# Supplementary material for: Non-invasive measurements of blood glucose levels by time-gating mid-infrared optoacoustic signals
Source: Nat Metab. 2024 Mar 27;6(4):678–86. doi: 10.1038/s42255-024-01016-9 (PMC11052715; doi:10.1038/s42255-024-01016-9)
Supplement: Supplementary file 2 — Reporting Summary [file 42255_2024_1016_MOESM2_ESM.pdf]

nature portfolio

Corresponding author(s): Vasilis Ntziachristos, Miguel A. Pleitez

Last updated by author(s): 2024.14.01

Reporting Summary

Nature Portfolio wishes to improve the reproducibility of the work that we publish. This form provides structure for consistency and transparency in reporting. For further information on Nature Portfolio policies, see our Editorial Policies and the Editorial Policy Checklist.

Statistics

For all statistical analyses, confirm that the following items are present in the figure legend, table legend, main text, or Methods section.

n/a	Confirmed	
☐	☒	The exact sample size (*n*) for each experimental group/condition, given as a discrete number and unit of measurement
☐	☒	A statement on whether measurements were taken from distinct samples or whether the same sample was measured repeatedly
☒	☐	The statistical test(s) used AND whether they are one- or two-sided<br>*Only common tests should be described solely by name; describe more complex techniques in the Methods section.*
☒	☐	A description of all covariates tested
☒	☐	A description of any assumptions or corrections, such as tests of normality and adjustment for multiple comparisons
☒	☐	A full description of the statistical parameters including central tendency (e.g. means) or other basic estimates (e.g. regression coefficient) AND variation (e.g. standard deviation) or associated estimates of uncertainty (e.g. confidence intervals)
☒	☐	For null hypothesis testing, the test statistic (e.g. *F*, *t*, *r*) with confidence intervals, effect sizes, degrees of freedom and *P* value noted<br>*Give P values as exact values whenever suitable.*
☒	☐	For Bayesian analysis, information on the choice of priors and Markov chain Monte Carlo settings
☒	☐	For hierarchical and complex designs, identification of the appropriate level for tests and full reporting of outcomes
☒	☐	Estimates of effect sizes (e.g. Cohen's *d*, Pearson's *r*), indicating how they were calculated

Our web collection on statistics for biologists contains articles on many of the points above.

Software and code

Policy information about availability of computer code

Data collection	For data collect data collection we used the commercially available softwares: GaGeScope Version 3.84.38 (Compuscope Driver, Dynamic Signals LLC, USA) and Matlab 2019b (Matlab, Mathworks, USA)
Data analysis	Data analysis was conducted using Matlab 2019b built-in functions (Matlab, Mathworks, USA) and the PLS_Toolbox 8.9.2, (Eigenvector Research Inc., Manson, WA, USA)

For manuscripts utilizing custom algorithms or software that are central to the research but not yet described in published literature, software must be made available to editors and reviewers. We strongly encourage code deposition in a community repository (e.g. GitHub). See the Nature Portfolio guidelines for submitting code & software for further information.

Data

Policy information about availability of data

All manuscripts must include a data availability statement. This statement should provide the following information, where applicable:

- Accession codes, unique identifiers, or web links for publicly available datasets
- A description of any restrictions on data availability
- For clinical datasets or third party data, please ensure that the statement adheres to our policy

The data that support the findings of this study are available from the corresponding authors upon reasonable request.

Research involving human participants, their data, or biological material

Policy information about studies with human participants or human data. See also policy information about sex, gender (identity/presentation), and sexual orientation and race, ethnicity and racism.

Reporting on sex and gender	N/A. No human participants or data was used in our study.
Reporting on race, ethnicity, or other socially relevant groupings	N/A. No human participants or data was used in our study.
Population characteristics	N/A. No human participants or data was used in our study.
Recruitment	N/A. No human participants or data was used in our study.
Ethics oversight	N/A. No human participants or data was used in our study.

Note that full information on the approval of the study protocol must also be provided in the manuscript.

Field-specific reporting

Please select the one below that is the best fit for your research. If you are not sure, read the appropriate sections before making your selection.

☒ Life sciences ☐ Behavioural & social sciences ☐ Ecological, evolutionary & environmental sciences

For a reference copy of the document with all sections, see nature.com/documents/nr-reporting-summary-flat.pdf

Life sciences study design

All studies must disclose on these points even when the disclosure is negative.

Sample size	No sample-size calculation was performed. Sample size was defined by sample availability based on our ethical guidelines and regulations governing the use of animals in research—indicating that the use the minimum number of animals necessary to obtain reliable results.
Data exclusions	No data was excluded from analysis.
Replication	All experiments presented here were successfully repeated independently at least ten times with similar results. Two experiments per day were performed independently for 5 consecutive days.
Randomization	The experiments were not randomized. The mice used in this research were selected from the same group regarding age, weight, genetic background. However, the imaged areas and measured points were arbitrary selected on each independent measurement.
Blinding	The investigators were not blinded to allocation during experiments and outcome assessment.

Reporting for specific materials, systems and methods

We require information from authors about some types of materials, experimental systems and methods used in many studies. Here, indicate whether each material, system or method listed is relevant to your study. If you are not sure if a list item applies to your research, read the appropriate section before selecting a response.

Materials & experimental systems

n/a	Involved in the study	
☒	☐	Antibodies
☒	☐	Eukaryotic cell lines
☒	☐	Palaeontology and archaeology
☐	☒	Animals and other organisms
☒	☐	Clinical data
☒	☐	Dual use research of concern
☒	☐	Plants

Methods

n/a	Involved in the study	
☒	☐	ChIP-seq
☒	☐	Flow cytometry
☒	☐	MRI-based neuroimaging

Animals and other research organisms

Policy information about studies involving animals; ARRIVE guidelines recommended for reporting animal research, and Sex and Gender in Research

Laboratory animals	Female Athymic nude-Foxn1nu mice (Envigo, Germany) were selected for the glucose tolerance tests. The study utilized mice aged 4-8 weeks.
Wild animals	No wild animals were used in this study.
Reporting on sex	Female Athymic nude-Foxn1nu mice (Envigo, Germany) were selected for the glucose tolerance tests.<br>They are easier to handle compared to male in the cages.
Field-collected samples	No field-collected samples were used in this study.
Ethics oversight	All mouse experiments were performed according to the guidelines of the committee on Animal Health Care of Upper Bavaria, Germany.

Note that full information on the approval of the study protocol must also be provided in the manuscript.

Plants

Seed stocks	Not available.
Novel plant genotypes	Not available.
Authentication	Not available
